# Five ways to find a parasite: comparison of five molecular assays for the detection of *Strongyloides* spp.

**DOI:** 10.1128/jcm.00137-26

**Published:** 2026-10-12

**Authors:** Juciliane Haidamak, Huan Zhao, Matthew R. Watts, Paul F. Horwood, Andrew R. Greenhill, Polydor Ngoy Mutombo, Rodrigue Mintsa Nguema, Rebecca Ford, Geraldine Masiria, Tobias Maure, Nichola Kotale, William Pomat, Natasha Collinson, Severine Navarro, Richard S. Bradbury, Catherine A. Gordon

**Affiliations:** 1 QIMR Berghofer, Mucosal Immunology Lab 56362 , Brisbane, Queensland, Australia; 2 James Cook University, Public Health and Tropical Medicine 8001, Townsville, Queensland, Australia; 3 Centre for Infectious Diseases and Microbiology, Institute of Clinical Pathology and Medical Research – New South Wales Health Pathology and Sydney and Infectious Diseases Institute, Westmead Hospital, University of Sydney 198104, Sydney, New South Wales, Australia; 4 James Cook University, College of Science and Engineering 91846, Cairns, Queensland, Australia; 5 Federation University, Microbiology Research Group, Institute of Innovation, Science and Sustainability 170469, Churchill, Victoria, Australia; 6 National Centre for Naturopathic Medicine, Southern Cross University 4571, Lismore, New South Wales, Australia; 7 National Center for Scientific and Technological Research, Libreville, Gabon; 8 Papua New Guinea Institute of Medical Research 116896, Goroka, Eastern Highlands Province, Papua New Guinea; 9 QIMR Berghofer, Applied Tropical and Molecular Parasitology Lab 56362 , Brisbane, Queensland, Australia; 10 The University of Queensland, School of Medicine 95561, Brisbane, Queensland, Australia; 11 Centre for Tropical Health and Emerging Diseases 589133 , Queensland, Australia; Mayo Clinic Minnesota, Rochester, Minnesota, USA

**Keywords:** *Strongyloides stercoralis*, *Strongyloides fuelleborni*, strongyloidiasis, qPCR, Papua New Guinea, PNG, Gabon

## Abstract

Strongyloidiasis, caused by nematodes of the genus *Strongyloides*, is a neglected tropical disease with significant public health implications. Accurate diagnostics are essential for disease surveillance and control, yet traditional stool-based and serological methods have limitations in sensitivity and specificity. Molecular assays offer an alternative diagnostic approach. In this study, we compared four probe-based real-time PCR (qPCR) assays and one loop-mediated isothermal amplification (LAMP) assay using human fecal DNA samples from Gabon and Papua New Guinea with both *Strongyloides stercoralis* and *Strongyloides fuelleborni* infections. Our results revealed discordance between assays, with no single primer set identifying all positive samples across all assays. One assay was identified as specific for *S. stercoralis* only, while other assays amplify other *Strongyloides* spp. This may impact the specificity of molecular diagnostics in epidemiological surveys, particularly with increasing identifications of *S. fuelleborni* infections in humans from Asia and New Guinea. Of the assays examined, four were probe-based qPCR assays and a LAMP assay. On sequencing positive amplicons, enteric bacterial sequences were identified for two assays. However, subsequent testing and BLAST results identified that including a probe, as the assays were designed, would increase specificity, so that the bacterial sequences would not be amplified. These findings provide a context for the interpretation of results from various *Strongyloides* molecular assays.

**IMPORTANCE:**

Strongyloidiasis is an underdiagnosed neglected parasitic infection that poses a growing challenge for public health surveillance and control, particularly in regions where multiple *Strongyloides* species exist and clinical case detection is challenging. Molecular assays are increasingly used as reference diagnostic tools, yet their performance and species specificity have not been systematically evaluated across diverse endemic settings. In this study, we tested the hypothesis that commonly used molecular assays differ in their ability to accurately and consistently detect and discriminate *Strongyloides stercoralis* and *Strongyloides fuelleborni* in human fecal samples. By comparing five molecular assays using samples from Gabon and Papua New Guinea, we demonstrate discordance between tests, including species-dependent detection biases. These findings provide evidence that molecular diagnostics may misrepresent species distribution. Our results highlight the necessity for awareness of target species specificity and for regionally validated molecular tools to ensure the reliable interpretation of assay results.

## INTRODUCTION

Strongyloidiasis is a potentially fatal parasitic disease caused by infection with nematodes of the genus *Strongyloides,* with an estimated 614 million people infected worldwide (1, 2). Strongyloidiasis was recognized as a soil-transmitted helminthiasis (STH) by the WHO in 2020 (3); however, the most widely used diagnostic for STH, the Kato Katz (KK) microscopy technique, is not viable for detection of *Strongyloides stercoralis* (4, 5). Thus, strongyloidiasis has been largely ignored in national control programs and mass drug administration (MDA) activities in endemic areas (4, 6). There is also evidence indicating that *S. stercoralis* may be a zoonosis, further complicating our understanding of transmission and control (7–9).

*Strongyloides fuelleborni* is a zoonotic species primarily of non-human primates in Africa and Asia, which is passed as eggs in stool rather than larvae. This may lead to misdiagnosis due to the morphological similarity of these eggs to larvated hookworm in human feces (5). Until recently, a unique sub-species, named “*S. fuelleborni* subsp. *kellyi,*” was thought to occur on the island of New Guinea, but this has recently been identified as representing human-to-human infection of the Asian clade of *S. fuelleborni* (10–13). Recent molecular analysis has indicated that the Asian and African clades of *S. fuelleborni* may represent separate species (14). Previous work by this group has also identified human infections with another undescribed *Strongyloides* species, closely related to *S. ransomi* of pigs (12, 15).

Diagnostics are critical for control programs to understand the burden of disease and elucidate epidemiology and in clinical practice for individual diagnosis and screening due to the potential for severe disease sequelae in immunocompromised individuals (16). Molecular methods are particularly relevant to the diagnosis of strongyloidiasis, as serology may be negative in immunocompromised patients with patent infections, and molecular approaches are a more sensitive diagnostic approach than traditional microscopy (5). Unlike culture-based methods, they have the added advantage of being possible to perform on preserved samples months after collection. Comparisons of test performance often group PCR tests together; however, each molecular test is different and has unique diagnostic characteristics. These characteristics include analytical sensitivity and specificity, target sequence copy number, species or genotype coverage, and tolerance to inhibitors—particularly important for stool samples, which are known to contain high concentrations of potential inhibitors (17). Furthermore, consideration needs to be given to pre-analytical processing factors and their impact on test performance, including the methods of stool preservation and DNA extraction employed (18–20). Analytical variables also influence assay efficacy, including the quality and storage of buffers, enzymes, primers, and probes, as well as the PCR cycler employed and whether recommended PCR cycling conditions and cut-off values were adhered to (5). There are a number of available molecular assays for the detection of *Strongyloides* spp. targeting nuclear repeat sequences or ribosomal genes (21–25). Of these, only one has been validated for clinical diagnostic use (23), while the remaining assays have primarily been applied in research or epidemiologic contexts rather than routine clinical diagnosis.

We chose to compare the most commonly employed DNA-based *Strongyloide*s assays to determine their relative sensitivity and specificity for both *S. stercoralis* and *S. fuelleborni* in stool. Human fecal DNA samples collected from Gabon in West Africa and Papua New Guinea (PNG) in Oceania, chosen for their geographical separation and the presence of multiple human-infecting *Strongyloides* spp., were used to directly compare four real-time PCR (qPCR) and one LAMP assay.

## MATERIALS AND METHODS

### Sample collection

#### Papua New Guinea

A 1-year longitudinal study was performed during 2018–2019 in the Eastern Highlands Province of PNG. Stool samples were collected from mothers and infants. Mothers submitted freshly collected stool samples at one or two time points soon after giving birth, while they were requested to submit stool samples from infants directly after birth and at each subsequent visit to the medical center within the 12 months of the study. A total of 196 stool samples were collected from infants and their mothers: 164 stool samples from 19 infants and 32 stool samples from their mothers. Due to the COVID-19 pandemic, stool samples remained at PNG IMR, frozen at −80°C, until they were able to be shipped to QIMR Berghofer, Australia, in 2023 for DNA extraction and subsequent qPCR. Samples were thawed once at the time of DNA extraction. Extracted DNA was stored at −80 °C until being thawed for PCR. No other parasitological examinations were performed on these samples.

#### Gabon

Participants were recruited by convenience sampling from seven villages located in the Ngounie Province of Gabon during 2023 (26). A total of 241 stool samples were collected, with one sample taken from each participant. These samples were preserved separately in 70% ethanol and 10% formalin and immediately shipped to Australia. Upon arrival, the 70% ethanol preserved samples were stored at room temperature for 3 months prior to DNA extraction. DNA extracts were stored at −80°C and thawed and refrozen twice for other analyses prior to being sent from JCU to QIMR Berghofer for qPCR. These samples were also examined microscopically using the formalin ethyl acetate concentration technique (FECT) (26).

### DNA extraction

#### Papua New Guinea

DNA was extracted at QIMR Berghofer using QIAamp DNA Mini Kit (Qiagen, Hilden, Germany), following the manufacturer’s protocol, with the exception of a pre-extraction step as follows. Stool (~200 mg) was placed into 2 mL screw cap tubes (Starstedt), along with 500 µL of ROSE buffer, 15 µL of proteinase K, and 500 µL of 0.5 mm silica/zirconia beads (Daintree Scientific, St. Helens, Australia). The tubes were placed into a Precelly’s homogenizer and homogenized at 6,500 rpm for 40 s. The tubes were then placed into a heat block at 95°C for 5 min before being centrifuged at 4,000 × *g* for 3 min. Supernatant was then transferred to a new 1.5 mL tube, and the manufacturer’s protocol was followed from step 4. The extracted fecal DNA was tested for quality and quantity using a NanoDrop 2000 (Thermo Fisher Scientific). DNA samples with a total concentration of more than 10 ng/µL and a 260/280 ratio between 1.6 and 2.10 were used as qPCR templates. DNA samples that did not meet those requirements were re-extracted. DNA was stored at −20°C until required for further analysis.

#### Gabon

DNA was extracted at JCU using a PowerSoil Pro kit (Qiagen, Hilden, Germany), following the manufacturer’s protocol with minor modifications. Specifically, prior to extraction, stool samples (*n* = 241; ~200 mg each) were centrifuged at 8,000 × *g* for 60 s to remove excess ethanol. Milli-Q water was then added to the samples and kept at 4°C overnight. After washing, the samples were centrifuged again at 8,000 × *g* for 60 s, and the supernatant was decanted. The bead-beating step was performed for 1 min on a FastPrep-24 5G Instrument (MP Biomedicals). DNA was eluted in 100 μL of solution C6 and stored at −20°C until required for further analysis.

### Probe-based real-time PCR (qPCR) and LAMP

All extracted DNA samples from each study location were subjected to four qPCR assays and one LAMP assay for detection of *Strongyloides* spp. (Table 1) (21–25). Primer and probe concentrations, assay reagents, and cycling conditions for all assays can be found in File S1. In all qPCR assays, 2 µL of 1:5 diluted DNA was used as the template, while 5 µL was used in the LAMP assay. Positive controls of DNA extracted from *S. stercoralis* (human) and *S. fuelleborni* (Guinea baboon, *Papio papio*) infected feces (provided by one of the authors; RSB), and positive controls of synthetic genes (gBlocks; IDT) in a 1:10–1:1,000,000 standard dilution series were used in each qPCR and LAMP run (File S1). Three no-template controls (NTC) were also run in triplicate for all runs. *Parastrongylus trichosuri* DNA derived from Australian common brushtail possum (*Trichosurus vulpecula*) feces, and DNA isolated from bacterial cultures of *Segatella copri* (formerly *Prevotella copri*) and *Mediterraneibacter gnavus* were used as a control in one run for each assay. qPCRs were run at both QIMRB and JCU.

**TABLE 1:** Primer and probes utilized in this study

Paper	Target		Primer name		Primer/probe sequence	Amplicon size (bp)
Llewellyn^*a*^ (22)	SSU rRNA			Forward 5′−3′	GGGCCGGACACTATAAGGAT ^c^	471
				Reverse 5′−3′	TGCCTCTGGATATTGCTCAGTTC	
				Probe 5′−3′ Cy 5	ACACACCGGCCGTCGCTGC	
Verweij^*a*^ (23)	*18S rRNA*		Stro18S-1530F	Forward 5′−3′	GAATTCCAAGTAAACGTAAGTCATTAGC	101
			Stro18S-1630R	Reverse 5′−3′	TGCCTCTGGATATTGCTCAGTTC	
			Stro18S-1586T	Probe 5′−3′ FAM	ACACACCGGCCGTCGCTGC	
Pilotte^*a*^ (24)	Repeat			Forward 5′−3′	CGCTCCAGAATTAGTTCCAGTT	138
				Reverse 5′−3′	GCAGCTTAGTCGAAAGCATAGA	
				Probe 5′−3′ FAM	ACAGTCTCCAGTTCACTCCAGAAGAGT	
Chankongsin^*a*^ (21)	*28s rRNA*			Forward 5′−3′	GCG AAC AAG TAC TGT GAA GGA AAA TTG	92
				Reverse 5′−3′	TGG CTC TGT ATG CTT CCA TCG T	
				Probe 5′−3′ HEX	ACG TCC TCT TTA ACT CTC TCT CCG G	
Watts^*b*^ (25)	*28s rRNA*		F3	Forward outer	GTGTAGGCTGGCGTAGT	184
			B3	Reverse outer	TTTCAATTTTAGCTTAGGACC	
			FIP	Internal forward	GCTACTATCACCAAGATCTGCAC-GCATTGAAGGTTATAAGCGTAAG;	
			BIP	Internal reverse	ACACAAGTGAGAATCTTGTGGACCTAACTCACAGTCAAATGATGT	
			LB	Loop primer	CGAAGTGGAAAAGGGTTTCACG	

^*a*^ Sequencing was performed using forward and reverse primers.

^*b*^ Sequencing was performed using forward and reverse outer primers.

^*c*^ Altered from forward primer of Verweij *18S rRNA.*

A human cytochrome B gene qPCR was used to detect inhibition, using previously published primers and conditions (27).

### SYBR Green qPCR and conventional PCR

The 18 DNA samples positive by the Chankongsin qPCR were also tested using a SYBR Green assay with the Chankongsin forward and reverse primers only. For the SYBR Green assay, a 10 µL reaction mixture was prepared containing 200 nM of forward and reverse primers, SYBR Green Master Mix (Applied Biosystems), Milli-Q water, and 2 µL of template DNA. The cycling conditions were the same as for the probe-based assay with a melt curve added at the end (Table S1). The SYBR assay was performed on the CFX 384 (Bio-Rad).

All qPCR assays were also run as a SYBR Green assay using the above conditions, with 2 µL of DNA isolated from bacterial cultures of *S. copri* and *M. gnavus* used as a control in one run for each assay.

For conventional PCRs, a reaction mix of 18 µL was made containing 200 nM of forward and reverse primers, 2 mL of buffer, 1.0 mM MgCl_2_, 800 mM of dNTPs, 1.25 units of Gold AmpliTaq (Applied Biosystems, Foster City, CA), Milli-Q water, and 2 µL of template DNA. The PCR was performed on a T100 thermocycler (Bio-Rad). PCR products were resolved on a 2% agarose gel by electrophoresis. Gels included SYBR Safe (Thermo Fisher Scientific) DNA dye. Gels were examined and images taken using a blue light transilluminator using an E-box (Vilber).

### Sequencing

Amplicons were sequenced using BigDye Terminator v3.1 Cycle Sequencing chemistry (Applied Biosystems, Thermo Fisher Scientific) and analyzed on an ABI capillary sequencer at QIMR Berghofer Medical Research Institute (Brisbane, Australia). For LAMP positives, only the outer primers for the LAMP assay and forward and reverse primers for qPCR were used in sequencing reactions. Sequencing reactions contained 6 pmol of the appropriate primer and 5 ng of PCR product per 100 base pairs (bp) of expected amplicon (28).

Where amplicons did not sequence or resulted in poor quality sequences, they were re-sequenced once more.

### Primer nucleotide BLAST

After seeing the results of sequencing the amplicons, the primers and probes for each qPCR assay and the outer primer of the LAMP assay were BLASTed against the NCBI GenBank core nucleotide database (core_nt) five times: first, with no restrictions; second, with *Strongyloides* spp. excluded; third, restricted to *S. copri* (formerly *P. copri*); fourth, restricted to *Anaerostipes hadrus*; and fifth, restricted to *Mediterraneibacter gnavus*.

### Statistical and bioinformatic analysis

Raw sequences were analyzed in Geneious Prime (version 2024.0.7). Sequences were trimmed and contigs assembled using *de novo* assembly. Discordant base calls were also examined and rectified as required. Consensus sequences were extracted from each contig and then BLASTed against the NCBI GenBank nucleotide collection (nr/nt) to determine the species.

SAS software (version 9.4) was used for all other analysis. Ninety-five percent confidence intervals (95% CI) were calculated using standard formulae based on the binomial distribution (prevalence) of each assay, and Cohen’s kappa was used to assess diagnostic agreement between the different molecular assays. As molecular tests are not reference standards, sensitivity and specificity were not calculated; instead, comparisons were calculated as positive percentage agreement (PPA) and negative percentage agreement (NPA), based on the U.S. Food and Drug Administration (FDA) guidelines (29). Negative predictive value (NPV) and positive predictive value (PPV) were also calculated in SAS. For these calculations, two different denominators were used. The first was a composite standard, where all molecular tests were positive, and the second was an operational consensus standard, where DNA extracts were positive by at least two of the five assays.

GraphPad Prism (Dotmatics) was used to graph Ct scores from each molecular assay, while R Studio (version 4.4.1) was used to create Euler diagrams.

## RESULTS

### Prevalence

**Fig 1:**
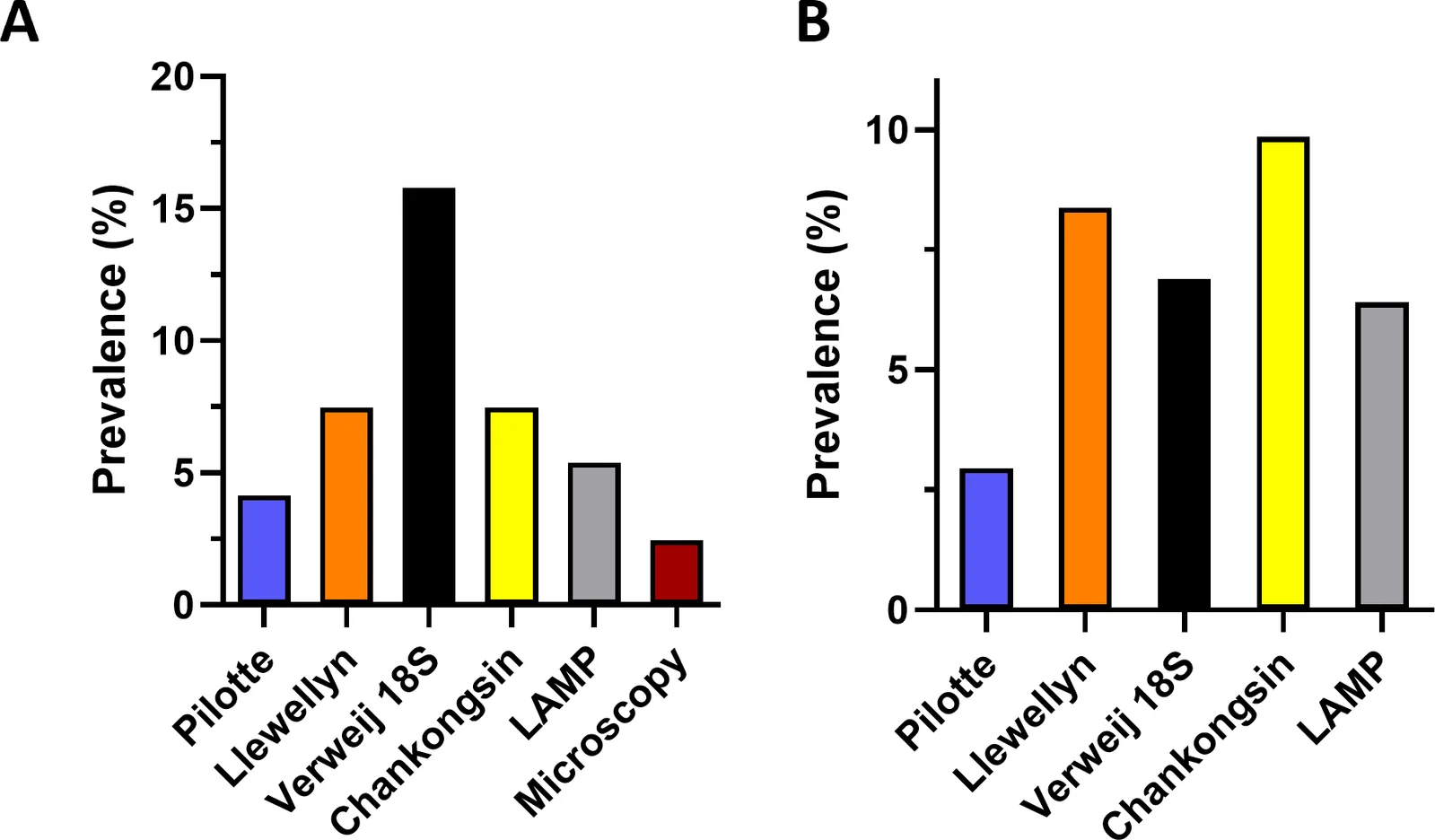
Prevalence of *Strongyloides* species as detected by five molecular diagnostic assays in fecal samples from (**A**) Gabon (*n* = 241) and (**B**) PNG (*n* = 196).

**Fig 2:**
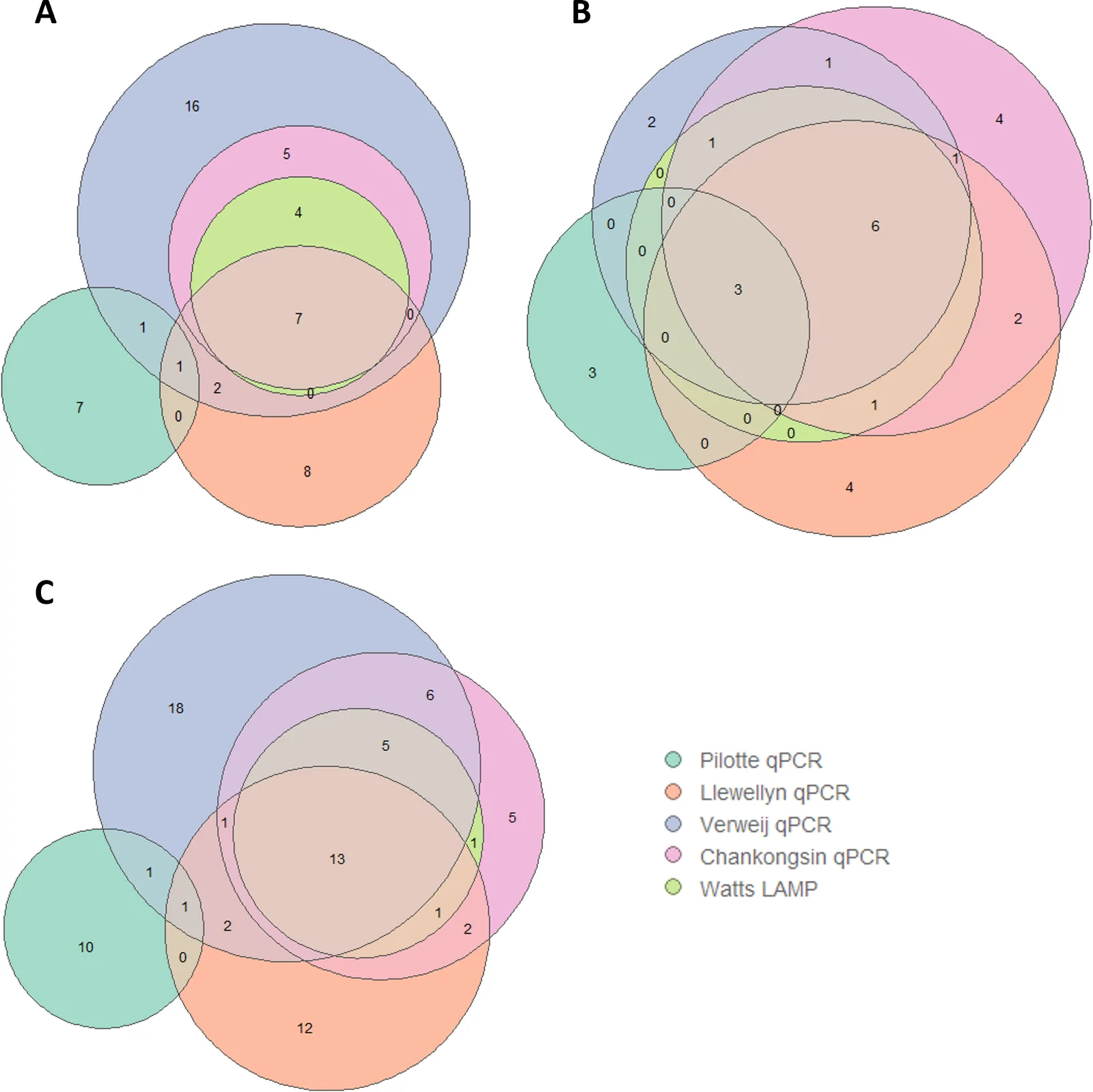
Venn diagram showing results of five molecular diagnostic assays detecting *Strongyloides* species on human fecal samples. (**A**) Assay results for samples from Gabon, (**B**) assay results from PNG, and (**C**) combined assay results for both Gabon and PNG.

The highest detection rate by any assay for the Gabon samples was identified with the Verweij qPCR (15.8%; 95% CI 14.4–24.5), followed by Chankongsin qPCR (7.5%; 95%CI 4.1–10.8) and the Llewellyn qPCR (7.5%; 95% CI 4.1–10.8) (Fig. 1). In PNG, the highest detection rate was identified with the Chankongsin qPCR assay (9.9%; 95%CI 5.7–13.9), followed by Llewellyn qPCR (8.4%; 95%CI 4.5–12.2) and Verweij qPCR (6.9%; 95%CI 3.3–10.4) (Fig. 1). Only five samples (2.07%; 95%CI 0.26–3.89) were positive by microscopy in the Gabon cohort, with four identified morphologically as *S. fuelleborni* and one as *S. stercoralis*. No other parasitological diagnostics were performed on the PNG samples. One of the microscopy-positive samples was not positive by any of the qPCR assays, while none of the remaining four samples were positive by all assays (Fig. 2). The Pilotte qPCR only identified the single *S. stercoralis* infection; two samples were positive by three qPCR assays, and the remaining microscopy-positive sample was positive by only one assay—the Verweij qPCR.

### Comparison of molecular methods

The assays showed better overall percentage agreement when applied to the PNG samples (Fig. 2). The full table of positives by any assay for Gabon and PNG studies can be found in Tables S1 and S2. Notably, in the Gabon cohort, there were 16 samples that were positive via the Verweij assay that were not positive by any other assay. Additionally, in Gabon, there were eight samples positive only by the Llewellyn assay, and seven positive only by the Pilotte assay. In the PNG cohort, the agreement was better, with no large numbers of samples positive only by one assay. The Pilotte assay identified three positives that were not positive by any other assay, the Llewellyn assay identified four, and Chankongsin also identified four positives that were not positive by any other assay.

**Fig 3:**
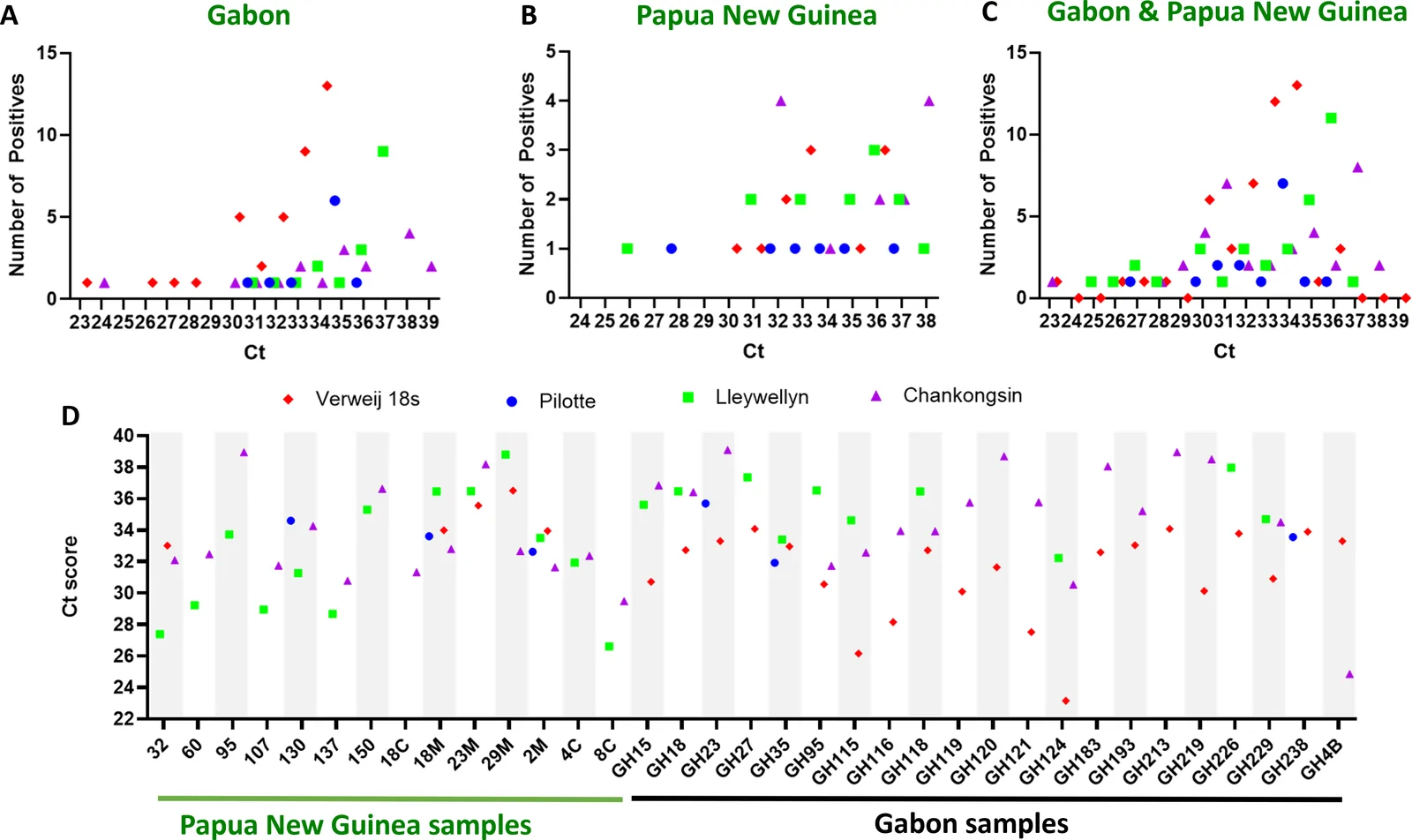
Graphical representation of cycle threshold (Ct) mean average of three replicate scores for each positive sample in four qPCR assays detecting *Strongyloides* species in human fecal samples. (**A**) Ct results for Gabon samples. (**B**) Ct results for PNG samples. (**C**) Ct results for the combined Gabon and PNG sample cohorts. (**D**) Ct scores for samples positive by at least two qPCR assays.

When comparing average cycle threshold (Ct) scores of positive samples, there was a general spread from 24 to 39 (Fig. 3A through C). There is an obvious cluster of a high number of positives by the Verweij assay that cluster around Ct 33–34 in the Gabon samples. When examining Cts for samples positive by at least one other qPCR assay, the Verweij qPCR had consistently lower Cts than those of the other assays in the Gabon samples (Fig. 3D). In the PNG samples, it was more mixed, with the Llewellyn qPCR and Chankongsin qPCR having generally lower Cts than other assays. This was reversed in Gabon, where the Chankongsin qPCR, in particular, exhibited higher Cts.

### Kappa

**Fig 4:**
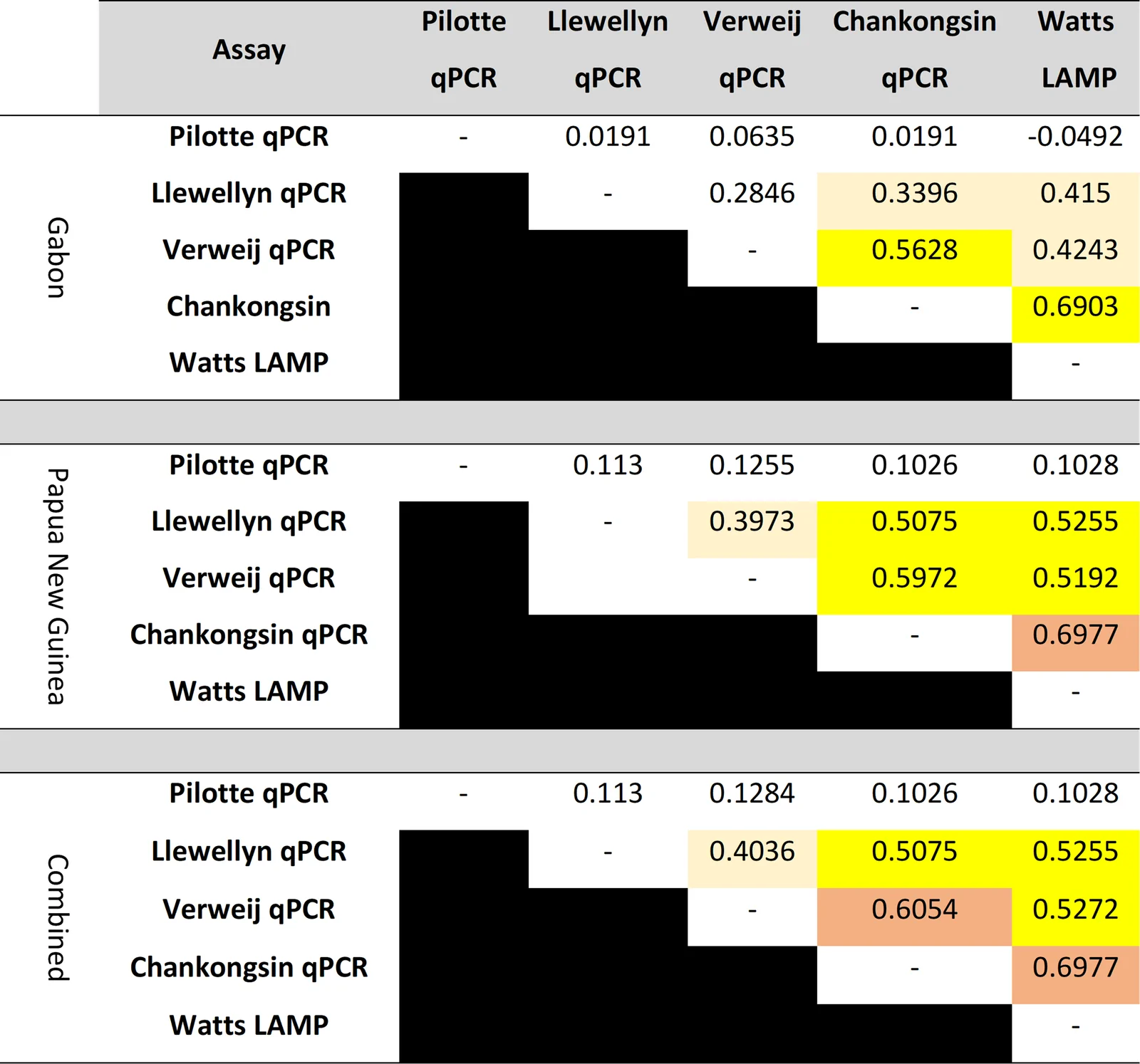
Kappa correlation testing results for five molecular diagnostic assays detecting *Strongyloides* species performed on human fecal samples from Gabon, PNG, and the combined cohort.

Comparison of the five assays showed little agreement between them, although there were slight differences depending on the country of derivation (Fig. 4).

Substantial agreement was observed between the Chankongsin qPCR and Watts LAMP assays and between the Verweij and Chankongsin qPCR assays when results from Gabon and PNG were combined, with similar patterns seen when the data sets were analyzed separately by country (Table 2).

**TABLE 2:** Microscopy results with corresponding qPCR and LAMP assay results showing Ct of positive qPCR assays

Species	Pilotte qPCR	Llewellyn qPCR	Verweij qPCR	Chankongsin qPCR	Watts LAMP
*S. stercoralis*	35.7			39.1	
*S. fuelleborni*					
*S. fuelleborni*			33.18		
*S. fuelleborni*		32.23		30.54	+
*S. fuelleborni*			33.30	24.87	+

### Positive percentage agreement, negative percentage agreement, PPV, and NPV

**Fig 5:**
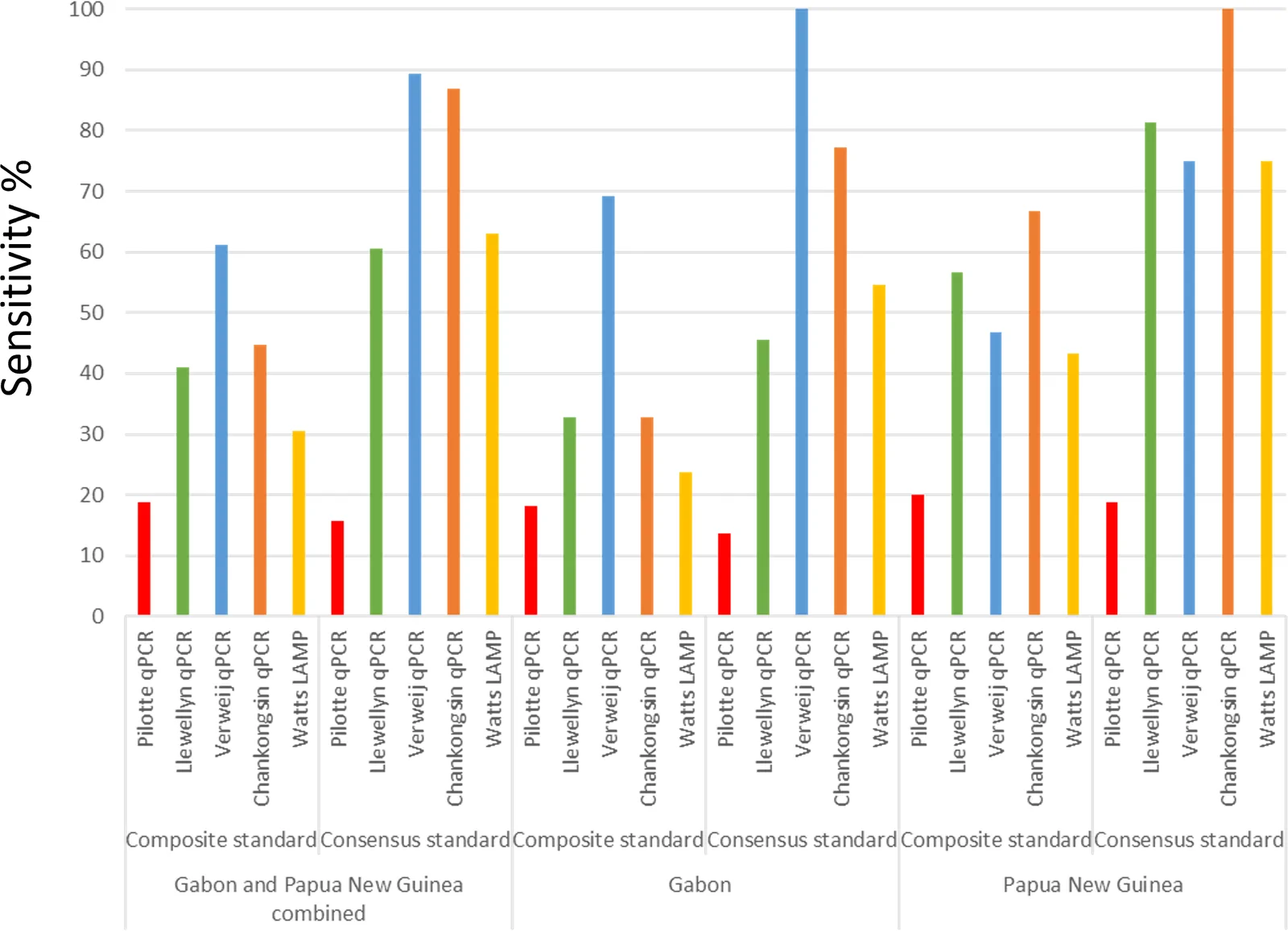
Positive percentage agreement of each assay, stratified by location and combined, compared against a standard of combination of results of all assays and a standard positive by at least two assays.

Overall NPA was high for all assays, with no assay below 90%, regardless of country cohort; however, NPA and PPV were not calculated for the composite standard in which all assays were positive. PPA varied across the different assays, reference standards, and between country cohorts (Fig. 5; Table S1). The lowest PPA was observed for the Pilotte qPCR across both cohorts and with both standards used. PPA was lower overall when the composite standard of all positive assays was employed. When the Gabon cohort was considered alone, the Verweij qPCR yielded the highest PPA, followed by the Chankongsin qPCR. In the PNG cohort, the Chankongsin qPCR demonstrated the highest PPA, followed by the Llewellyn assay. PPV was low overall for the Pilotte qPCR, Llewellyn qPCR, and Verweij qPCR, although this did vary slightly by country cohort. NPV was high across all assays and cohorts, at over 80% for all assays (Table S1).

### Sequencing

**Fig 6:**
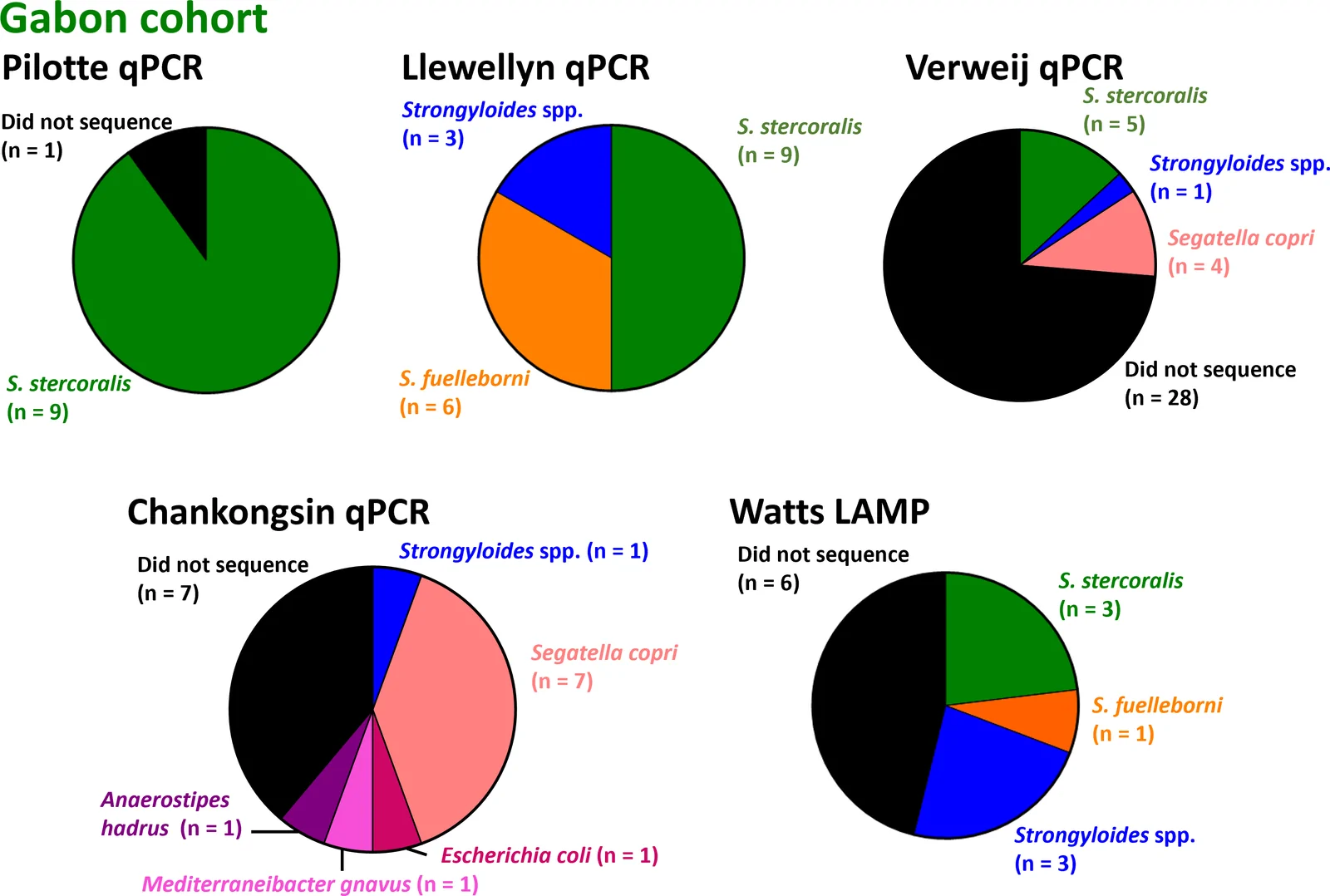
Sequencing results for Gabon cohort by five molecular diagnostic assays detecting *Strongyloides* species in human feces.

**Fig 7:**
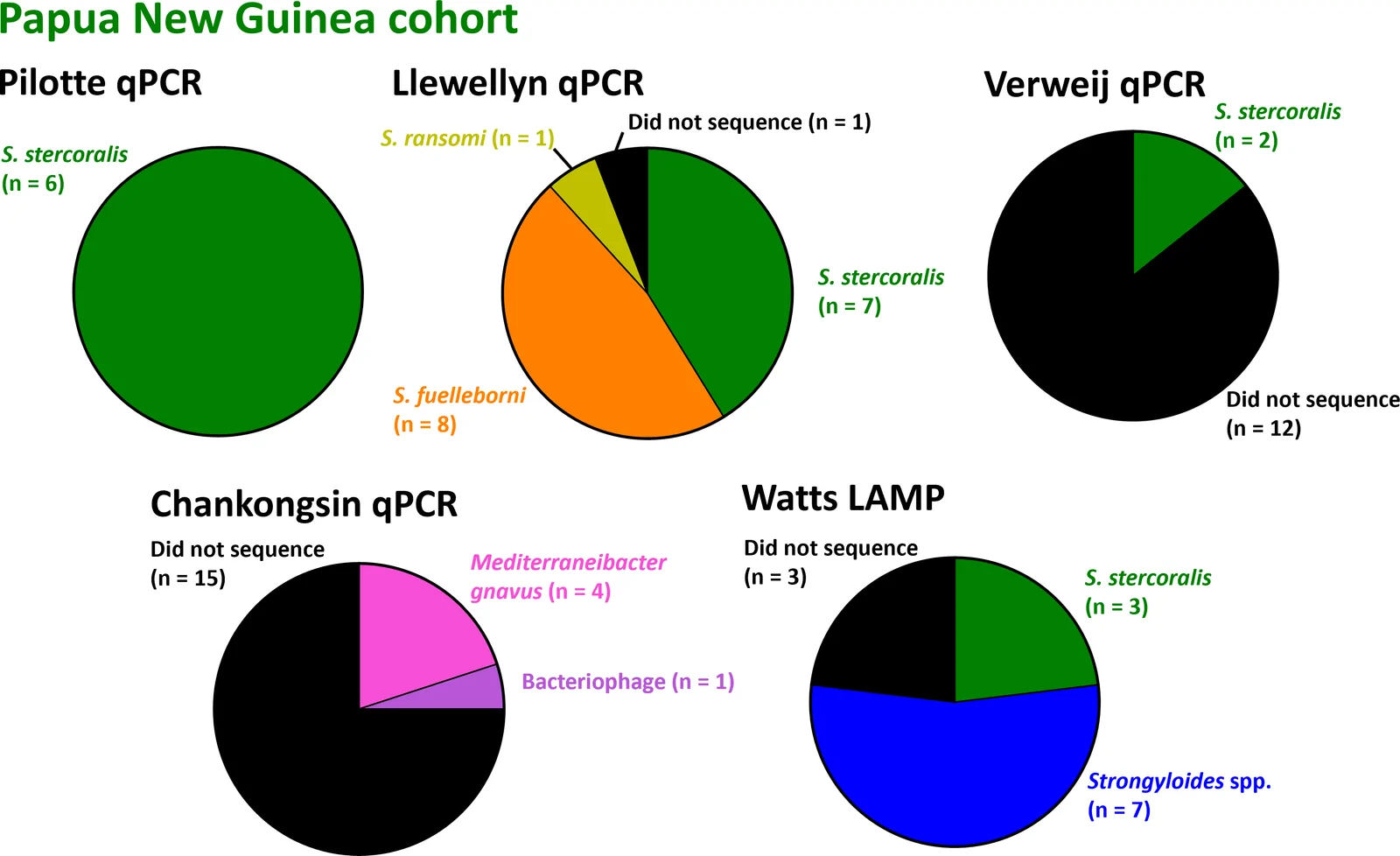
Sequencing results for PNG cohort by cohort by five molecular diagnostic assays detecting *Strongyloides* species in human feces.

Amplicons not yielding a band on the gel or a high-quality sequence on the first attempt were re-sequenced; not all amplicons were successfully sequenced. Only bands of the expected size were observed on gels. Both the Verweij and Chankongsin qPCRs had a low success rate for sequencing (Fig. 6 and 7). Full sequencing results can be found in Tables S2 and S3.

Without the specificity conferred by the assay probes, the Verweij and the Chankongsin primers returned enteric bacterial sequences from the Gabon cohort (*Segatella copri*, *Anaerostipes hadrus*, *M. gnavus*, and *Escherichia coli*) (Fig. 6; Table S3). Only one of the Chankongsin qPCR-positive samples in the Gabon cohort returned a match for a *Strongyloides* species, which was identified as *S. fuelleborni*.

In the PNG cohort, only the Chankongsin qPCR assay returned results for enteric bacteria (*M. gnavus*) and bacteriophage (Fig. 7; Table S2).

In both sample cohorts, the Pilotte qPCR returned sequences only for *S. stercoralis* and, when compared with the results of the other assays, did not amplify any *S. fuelleborni* positives. Notably, one sample positive by the Pilotte qPCR assay from the PNG cohort yielded a sequence by the Llewellyn assay with 100% identity (100% query cover) to *S. ransomi* when tested using nucleotide BLAST (BLASTn; Table S3). There were very high numbers of amplicons that did not sequence in both the PNG and Gabon samples using the Verweij and Chankongsin assays.

The LAMP assay returned positive results for *S. stercoralis* and *S. fuelleborni* on nucleotide BLASTn, but also returned results indicating that the sequence could have come from a number of different *Strongyloides* spp., including *S. stercoralis*, *S. ratti*, *S. ransomi*, *S. procyonis*, and *S. robustus* (Fig. 6 and 7; Tables S2 and S3).

### Off-target amplifications

Samples positive by the Chankongsin qPCR were run on a SYBR Green assay and a conventional PCR assay. A single peak was observed on the melt-curve analysis in the SYBR Green qPCR, and only single bands of expected size were observed in the conventional PCR assay (data not shown). All assays were negative when using *P. trichosuri* DNA as the template in both probe-based and SYBR Green assays. Only the Chankongsin primers amplified DNA of *M. gnavus* when run as a SYBR Green assay, and no assay amplified *S. copri*.

### Primer nucleotide BLAST

BLASTn analysis showed minimal off-target similarity for all assays, with low e-value matches observed against *S. copri*, *A. hadrus*, and *M. gnavus*, and all other hits exceeding e-values of 1 (Table S3). When unrestricted, Pilotte qPCR returned only *Strongyloides stercoralis* sequences, while Chankongsin primers matched exclusively *Strongyloides* spp. in the top 100 hits. Limited non-*Strongyloides* hits were observed for the Llewellyn and Verweij assays, including *Rhabditida* spp. and *P. trichosuri* (Table S4).

## DISCUSSION

We compared the results of five molecular diagnostics assays for the detection of *Strongyloides* species in human fecal samples from Gabon and PNG, containing both *S. stercoralis* and *S. fuelleborni* infections. Overall, there was poor correlation between results of each individual assay, with overall agreement better in the PNG samples compared to Gabon. For the purpose of this paper, the PNG samples were considered separately rather than as combined results from several samples collected longitudinally from each individual. These findings have implications for both individual patient diagnostics and population-level surveillance.

Discordance between assays may be due to the differences in collection and storage procedures, DNA extraction, the population cohorts examined, and possible geographic differences in *Strongyloides* spp. genotypes. The Gabon samples were fixed with 70% ethanol and shipped within 1 month of collection to Australia for DNA extraction with the Qiagen Powersoil Pro Kit, which is considered by many to be the optimal DNA extraction kit for stool samples (20, 30). These DNA samples were stored at −80°C and were thawed twice for less than 1 h, then refrozen, for other analyses prior to the analysis performed for this paper. In contrast, the PNG fecal samples were not fixed and instead frozen at −80°C, and there was a significant delay between collection and DNA extraction due to the COVID-19 pandemic. PNG samples were extracted with the Qiagen QIAmp DNA Kit and then stored at −20°C until required for further analysis. Samples fixed in ethanol may become dehydrated, which can reduce DNA extraction efficiency. However, to mediate this potential issue, the Gabon samples were rehydrated overnight immediately prior to DNA extraction.

The qPCR assays assessed had differing Ct cut-off values, as well as differences in primer and probe concentrations. The primer and probe concentrations were employed as described in the original primary descriptions of the assays. However, not all assays had a stated cut-off value. In this paper, we used the performance of a standard curve based on a dilution series of positive parasite DNA and the performance to non-template controls to help set the cut-off thresholds used. We also employed a human cytochrome *b* assay as an inhibition and DNA extraction control; however, inclusion of an exogenous internal amplification control at a defined concentration would have provided greater clarity as to whether the discordant results reflected low parasite burden, qPCR inhibition, or sub-optimal DNA extraction. Previous studies have employed synthetic strands of equine herpes virus DNA as such a control (22, 31). Due to discordant results, all positive amplicons were sequenced. Of the 16 Gabon samples that were positive by the Verweij assay but negative by other assays, most showed no detectable bands on conventional PCR using the Verweij forward and reverse primers, with bands of the expected size observed only in a subset of reactions; three of these sequenced as *S. stercoralis* and five as *S. copri*. Although limited BLAST similarity was observed for the Verweij reverse primer with *S. copri*, no amplification occurred when DNA from *S. copri* cultures or *P. trichosuri* was tested using the Verweij assay in either probe-based or SYBR formats (Table S3). Consistent with previous large-scale hospital and external quality assessment studies (32, 33), no evidence of non-specific amplification was observed, and the probe-based design further mitigates off-target binding. We therefore conclude that the observed disagreement is unlikely to be due to non-specific amplification.

Sequencing of products of the Chankongsin primers employed as a conventional PCR identified several enteric bacteria (*S. copri*, *A. hadrus*, and *M. gnavus*). Based on BLAST e-values and sequencing, we hypothesized that the assay correctly amplifies *Strongyloides* spp. but may also amplify background enteric bacteria, with any such amplification nullified by the *Strongyloides*-specific probe. Consistent with this, SYBR Green and conventional PCR of Chankongsin-positive samples showed no evidence of secondary targets. The primers did amplify *M. gnavus* DNA in SYBR format, confirming primer non-specificity, but this is mitigated by Taqman probe-based detection when the assay is employed as described by the original authors. In addition, one Gabon sample sequence demonstrated 100% identity to multiple *Strongyloides* spp. on BLAST, indicating that the assay is not specific to *S. stercoralis* and will amplify and detect other *Strongyloides* species (Table S3).

The inability to sequence some PCR products likely reflects their short amplicon lengths rather than poor analytical performance of the assays themselves (Table 1). The qPCR amplicons for the Chankongsin, Verweij, and Pilotte assays were 92–138 bp, which lies within the size range known to be challenging for Sanger sequencing, as the first 20–40 bp of a read are often low quality, leaving very short products and low-confidence sequence interpretation as a result (34). The LAMP and Llewellyn qPCR both have longer amplicons, 184 and 471 bp respectively, and returned a higher number of high-quality sequences when compared to the other assay, confirming that it is the short amplicon length causing the sequencing issues in the other assays. Additionally, removal of the Taqman probe for qPCR and inner primers for LAMP reduces assay specificity and sensitivity which also likely contributed to lower success in sequencing. Accurate species identification requires haplotyping of hypervariable regions, such as those in the *18S rRNA* and *cox1* genes, which have been used previously for *Strongyloides* genotyping (9).

Agreement among assays was higher in the PNG cohort, with the exception of the Pilotte qPCR, which also showed poor agreement in Gabon. This reflects its high specificity of this assay for *S. stercoralis sensu stricto* rather than poor assay performance. Sequencing indicated that all Pilotte-positive samples were all *S. stercoralis*, with one exception (the *S. ransomi*-like species from PNG), whereas samples positive by other assays included *S. fuelleborni* isolate. The Pilotte qPCR targets a non-coding tandem repeat region (24). Non-coding repeats are attractive targets for diagnostic qPCR due to their high copy number. They also demonstrate rapid divergence, which may be appealing for species-specific identification but, as demonstrated in this work, is less helpful when attempting to detect closely related pathogenic species using a single assay. The other molecular assays tested all target the same 18S nuclear rDNA unit, for which the range of estimated copies for nematodes varies between 56 and 323 per genome. Non-coding regions, such as that targeted by the Pilotte qPCR, tend to occur in higher abundance, although an estimate of copy number is not available for the Pilotte qPCR *S. stercoralis* repeat target.

A single sample, positive by all assays except the Verweij qPCR, returned sequences compatible with *S. ransomi* (Table S2), a parasite of pigs, in a human infant sample from PNG. Subsequent haplotyping analysis of this isolate has previously identified it as a unique lineage, related to the porcine parasite *S. ransomi* (12). This is consistent with *S. ransomi*-like *Strongyloides* found in human samples from PNG by enzyme electrophoresis 30 years ago, but at the time, this was assumed to reflect pig samples being submitted by participants (15). Furthermore, Dorriss et al. identified this haplotype in a PNG human sample containing *Strongyloides* eggs in 2002 (35). It is possible that porcine *S. ransomi* has infected humans in PNG and evolved over time into a novel, zoonotic *Strongyloides* species.

The Watts LAMP assay was originally designed based on *S. ratti* and targets a highly conserved region of the genome (*18S rRNA*). It is therefore no surprise that this assay detected both *S. Strongyloides* and *S. fuelleborni* species in both cohorts. However, the highly conserved nature of the region and small size (184 bp) meant that speciation was not possible based on the sequencing in this study. Ten samples from both cohorts returned high identity to a number of *Strongyloides* spp., including *S. procyonis*, *S. robustus*, and *S. ratti*, as well as *S. stercoralis* and *S. fuelleborni* (Tables S1 and S2).

All assays tested, with the exception of the Pilotte qPCR, yielded positive amplicons for both *S. stercoralis* and *S. fuelleborni*. These two species are known to infect both humans and non-human primates from Africa (9, 36–38). However, human infections have also recently been identified in human cases in Thailand (39), India (9, 37), and Bangladesh (40). The reported low prevalence in the literature of *S. fuelleborni* identified from humans in Asia compared to Africa may be attributed to a combination of lack of awareness and incorrect identification by microscopy, with eggs of *S. fuelleborni* being easily mistaken for morphologically similar hookworm eggs. The results of this study have demonstrated that *S. fuelleborni* infections may be misidentified as *S. stercoralis* using many commonly applied molecular diagnostic assays. This has important implications for PCR-based surveillance studies informing strongyloidiasis control programs for (41). A recently developed duplex for *S. stercoralis* and *S. fuelleborni* seeks to improve this, but was not published at the time that the comparisons reported in this manuscript were performed. This species-specific duplex qPCR assay has been compared to the *Strongyloides* genus-specific Verweij qPCR assay, with the latter demonstrating higher sensitivity (41).

Treatment protocols for strongyloidiasis vary internationally, with empiric ivermectin treatment suggested by the U.S. Centers for Disease Control and Prevention (CDC) (42) for all refugees both overseas and onshore when emigrating to the USA, while in Australia and Europe, the preferred approach is serological testing followed by treatment of positive cases (43). While empiric ivermectin treatment for high-risk populations is understandable, this strategy partly reflects the inadequacy of current diagnostics rather than the absence of a need for definitive testing.

Serological tests are important for screening but may perform poorly in cases of early infection, where there is immunocompromise, and where there is heavy infection (5). It is also affected by cross-reactivity with other helminths (4). Furthermore, serology cannot differentiate current from recent infection, and some patients will retain elevated IgG antibodies even after 12 months following treatment (44, 45). Definitive and specific parasitological diagnosis remains important not only for individual patient management but also for surveillance, prevalence estimation, and measuring the impact of control efforts (5). In routine practice, traditional stool-based examinations can be relatively insensitive, with serial stool examinations required to increase sensitivity (5). However, microscopy-based tests and culture still have a diagnostic role, as a relatively large volume of stool is tested, they are cost-effective, and they have good sensitivity where there is a higher larval burden (4). Molecular tests provide greater flexibility with transportation of preserved specimens, allow for centralized batch testing, and can reduce the risk of laboratory-acquired infection (5). They also confirm current infection and can be multiplexed with other stool pathogens to streamline workflows. This benefits individual diagnosis, screening, and population-based epidemiological studies and treatment programs.

### Conclusion

This comparative evaluation of five molecular assays for *Strongyloides* detection in samples from Gabon and Papua New Guinea revealed substantial variability in test outcomes and species specificity between regions. Limited agreement in the detection of *S. stercoralis*, *S. fuelleborni*, and *S. ransomi*-like isolates by the majority of assays highlights the complexity of molecular diagnosis in settings where multiple *Strongyloides* species co-circulate. These findings underscore the importance of assay validation across diverse endemic settings and the need for awareness of assay specificity when interpreting results for individual diagnostics and surveillance and control programs.

## Data Availability

All data generated or analyzed during this study are included in this published article and its supplemental material.

## SUPPLEMENTAL MATERIAL

File S1 jcm.00137-26-s0001.docx Assay conditions and details on positive controls used.

Figure S1 jcm.00137-26-s0002.docx SYBR Green real-time PCR results for *Strongyloides*-positive samples by Chankongsin assay showing amplification with a single melt curve.

Figure S2 jcm.00137-26-s0003.docx Results of conventional PCR using real-time PCR *Strongyloides*-positive samples using the Chankongsin assay showing only single bands.

Table S1 jcm.00137-26-s0004.docx Positive percentage agreement, negative percentage agreement, NPV, and PPV of each assay stratified by location and combined compared against a composite standard (combination of results of all assays) and a consensus standard (positive by at least two assays).

Table S2 jcm.00137-26-s0005.docx Results of sequencing assay amplicons for stool samples from Papua New Guinea showing average Ct score, assay results (+/−), and result of sequencing analysis with the percentage identity and coverage (%/%).

Table S3 jcm.00137-26-s0006.docx Results of sequencing assay amplicons for stool samples from Gabon showing average Ct score, assay results (+/−), and result of sequencing analysis with the percentage identity and coverage (%/%).

Table S4 jcm.00137-26-s0007.docx e-values of primers and probes from each assay when restricted to (1) *Segatella copri*, (2) *Anaerostipes hadrus*, (3) *Mediterraneibacter gnavus*, (4) no restrictions, and (5) with *Strongyloides* excluded.

ASM does not own the copyrights to Supplemental Material that may be linked to, or accessed through, an article. The authors have granted ASM a non-exclusive, world-wide license to publish the Supplemental Material files. Please contact the corresponding author directly for reuse.
