# Supplementary material for: Five ways to find a parasite: comparison of five molecular assays for the detection of Strongyloides spp
Source: J Clin Microbiol. 2026 Oct 12;64(11):e00137-26. doi: 10.1128/jcm.00137-26 (PMC13753838; doi:10.1128/jcm.00137-26)
Supplement: Supplementary file 1 [file jcm.00137-26-s0001.docx]

**Supplementary file 1**: Assay conditions and details on positive controls used.

| **Cycling conditions used for qPCR and LAMP** | | | | | | | | | | | |
| --- | --- | --- | --- | --- | --- | --- | --- | --- | --- | --- | --- |
| **Initialisation** | | **Initialisation** | | **Denaturation** | | **Annealing** | | **Extension** | | **Cycle no.** | **Cycle cut-off for negative** |
| **Temp. (ᵒC)** | **Time (mins)** | **Temp. (ᵒC)** | **Time (mins)** | **Temp. (ᵒC)** | **Time (mins)** | **Temp. (ᵒC)** | **Time (mins)** | **Temp. (ᵒC)** | **Time (mins)** |  |  |
| 50 | 2:00 | 95 | 10:00 | 95 | 0:15 | 59 | 1:00 |  |  | 40 | 38 |
|  |  | 95 | 15:00 | 95 | 0:30 | 55 | 0:30 | 72 | 0:30 | 40 | 38 |
|  |  | 95 | 15:00 | 95 | 0:15 | 60 | 1:00 |  |  | 50 | 35 |
| 50 | 2:00 | 95 | 10:00 | 95 | 0:15 | 58 | 1:00 |  |  | 45 | 40 |
| 60 | 60:00:00 |  | | | | | | | | | |

|  | **Reagents and amounts used for qPCR and LAMP** | | | | | | | | | | | |  | |  | |  | |  | |  | |  | |  | |
| --- | --- | --- | --- | --- | --- | --- | --- | --- | --- | --- | --- | --- | --- | --- | --- | --- | --- | --- | --- | --- | --- | --- | --- | --- | --- | --- |
| **Assay** | | **[Ref]** | **GoTaq*** | **Primer** conc. (nM)** | **Probe*** conc. (nM)** | **DNA (µl)** | |  | | **Total volume** | |  | |  | |  | |  | |  | |  | |  | |  |
| Pilotte qPCR | | [1] | 8 | 100 | 100 | 2 | |  | | 15 | |  | |  | |  | |  | |  | |  | |  | |  |
| Llewellyn qPCR | | [2] | 8 | 100 | 100 | 2 | |  | | 15 | |  | |  | |  | |  | |  | |  | |  | |  |
| Verweij qPCR | | [3] | 8 | 100 | 100 | 2 | |  | | 15 | |  | |  | |  | |  | |  | |  | |  | |  |
| Chankongsin qPCR | | [4] | 8 | 800 | 200 | 2 | |  | | 15 | |  | |  | |  | |  | |  | |  | |  | |  |
| *GoTaq (Promega) | | |  |  |  |  | |  | |  | |  | |  | |  | |  | |  | |  | |  | |  |
|  | **Primers ordered from Integrated DNA Technologies | | | | | | | | | | | |  | |  | |  | |  | |  | |  | |  | |
|  | ***Probes ordered from Integrated DNA Technologies, all probes ordered as FAM | | | | | | | | | | | |  | |  | |  | |  | |  | |  | |  | |
|  | |  |  |  |  |  | |  | |  | |  | |  | |  | |  | |  | |  | |  | |  |
| **LAMP** | | **[Ref]** | **MasterMix*** | **FIP/BIP (pmol)** | **LB** | **F3/B3 (pmol)** | |  | | **DNA (µl)** | |  | |  | |  | |  | |  | |  | |  | |  |
| Watts LAMP | | [5] | 12.5 | 40 | 20 | **F4** | |  | | 5 | |  | |  | |  | |  | |  | |  | |  | |  |
| *Warm start colorimetric LAMP master mix (New England Biolabs) | | | | | | |  | |  | |  | |  | |  | |  | |  | |  | |  | |  | |

All qPCR samples were run in triplicate. If the standard deviation was less than 1 and could not be resolved by removing one of the replicates samples were re-run.

# Positive controls

*S. stercoralis* – DNA extracted from infected human faeces (Australian acquisition)

*S. fuelleborni* – DNA extracted from infected Guinea baboon (*Papio papio*) faeces (Gambia acquisition)

# gBlocks

| **Assay** | **Gene** | **Accession number gBlock based on** | **gBlock sequence** |
| --- | --- | --- | --- |
| Pilotte qPCR | Repeat | AY028262 | ACAGCTCTCACGCTCCAGAATTAGTTCCAGTTGAATAACAGTCTCCAGTTCACTCCAGAAGAGTTCCTATAATCCTAACTCAGCTCCAGTAAAGCAACAGTTTCCAACCCCTCACAAAAGAGCTTCTATGCTTTCGACTAAGCTGCAGTATAGGTACATCCTC |
| Llewellyn qPCR | SSU rRNA | STDRG18S | GGGCCGGACACTATAAGGATTGACAGATTGATAGCTCTTTCATGATTTAGTGGTTGGTGGTGCATGGCCGTTCTTAGTTCGTGGATATGATTTGTCTGGTTGATTCCGATAACGAGCGAGACGAGAGAGAGCGATAACAGGTCTGTGATGCCCTTAGATGTCCGGGGCTGCACGCGCGCTACAATGTAGTGATCATTATGTTCCTGTTTAGAGATAAATGGGTAAACATTGAAAACATTACGTAACTGGGAGTGAAAATTGCAATTATTTTTCATGAACGAGGAATTCCAAGTAAACGTAAGTCATTAGCTTACATTGATTACGTCCCTGCCCTTTGTACACACCGGCCGTCGCTGCCCGGAACTGAGCAATATCCAGAGGCA |
| Verweij qPCR | 18s rRNA | AF279916 | TCATGAACGAGGAATTCCAAGTAAACGTAAGTCATTAGCTTACATTGATTACGTCCCTGCCCTTTGTACACACCGGCCGTCGCTGCCCGGAACTGAGCAATATCCAGAGGCAGGAAGAGATGTAA |
| Chankongsin qPCR | 28s rRNA | LC259808 | GATAAACATCATCTAAGGCTAAATATTCCTATGAATCCGATAGCGAACAAGTACTGTGAAGGAAAATTGCAAAGTACTCCGGAGAGAGAGTTAAAGAGGACGTGAAACCGATACGATGGAAGCATACAGAGCCAGTTGCAC |
| Watts LAMP | 28s rRNA | DQ145710 | TATATGTGTAGGCTGGCGTAGTAATAGCATTGAAGGTTATAAGCGTAAGCTTAACTGGAGCTGTTATTAGTGCAGATCTTGGTGATAGTAGCAAATACACAAGTGAGAATCTTGTGGACCGAAGTGGAAAAGGGTTTCACGTCAACATCATTTGACTGTGAGTTAGTCGGTCCTAAGCTAAAATTGAAAAATTTTTATTCATATATAGC |

1.	Pilotte, N., et al., *Improved PCR-based detection of soil transmitted helminth infections using a next-generation sequencing approach to assay design.* PLoS Negl Trop Dis, 2016. **10**(3): p. e0004578.

2.	Llewellyn, S., et al., *Application of a multiplex quantitative PCR to assess prevalence and intensity of intestinal parasite infections in a controlled clinical trial.* PLoS Negl Trop Dis, 2016. **10**(1): p. e0004380.

3.	Verweij, J.J., et al., *Molecular diagnosis of Strongyloides stercoralis in faecal samples using real-time PCR.* Transactions of the Royal Society of Tropical Medicine and Hygiene, 2009. **103**(4): p. 342-346.

4.	Chankongsin, S., et al., *Strongyloides stercoralis prevalence and diagnostics in Vientiane, Lao People’s Democratic Republic.* Infectious Diseases of Poverty, 2020. **9**(1): p. 133.

5.	Watts, M.R., et al., *A loop-mediated isothermal amplification (LAMP) assay for Strongyloides stercoralis in stool that uses a visual detection method with SYTO-82 fluorescent dye.* The American Journal of Tropical Medicine and Hygiene, 2014. **90**(2): p. 306-311.
