# Supplementary material for: Five ways to find a parasite: comparison of five molecular assays for the detection of Strongyloides spp
Source: J Clin Microbiol. 2026 Oct 12;64(11):e00137-26. doi: 10.1128/jcm.00137-26 (PMC13753838; doi:10.1128/jcm.00137-26)
Supplement: Supplementary file 2 [file jcm.00137-26-s0002.docx]

**Supplementary figure 1: Sybr Green r**eal-time PCR results for *Strongyloides* positive samples by Chankongsin assay showing amplification with a single melt curve.
