# Supplementary material for: Five ways to find a parasite: comparison of five molecular assays for the detection of Strongyloides spp
Source: J Clin Microbiol. 2026 Oct 12;64(11):e00137-26. doi: 10.1128/jcm.00137-26 (PMC13753838; doi:10.1128/jcm.00137-26)
Supplement: Supplementary file 3 [file jcm.00137-26-s0003.docx]

**Supplementary figure 2:** Results of conventional PCR using real-time PCR *Strongyloides* positive samples using the Chankongsin assay showing only single bands.
