# Supplementary material for: Five ways to find a parasite: comparison of five molecular assays for the detection of Strongyloides spp
Source: J Clin Microbiol. 2026 Oct 12;64(11):e00137-26. doi: 10.1128/jcm.00137-26 (PMC13753838; doi:10.1128/jcm.00137-26)
Supplement: Supplementary file 4 [file jcm.00137-26-s0004.docx]

**Supplementary Table 1:** Positive Percentage Agreement, Negative Percentage Agreement, NPV, and PPV of each assay stratified by location and combined compared against a composite standard (combination of results of all assays) and a consensus standard (positive by at least two assays).

|  |  | **Assay** | **TP** | **FN** | **FP** | **TN** | **Sensitivity % (95% CI)** | **Specificity % (95% CI)** | **PPV %** | **NPV%** |
| --- | --- | --- | --- | --- | --- | --- | --- | --- | --- | --- |
| **Gabon and Papua New Guinea combined** | Composite standard | Pilotte qPCR | 16 | 69 | 0 | 359 | 18.82 (10.51 - 27.13) | NA | NA | 83.88 |
|  |  | Llewellyn qPCR | 35 | 50 | 0 | 359 | 41.18 (30.71 - 51.64) | NA | NA | 87.78 |
|  |  | Verweij qPCR | 52 | 33 | 0 | 359 | 61.18 (50.82 - 71.54) | NA | NA | 91.58 |
|  |  | Chankongsin qPCR | 38 | 47 | 0 | 359 | 44.71 (34.14 - 55.28) | NA | NA | 88.42 |
|  |  | Watts LAMP | 26 | 59 | 0 | 359 | 30.59 (20.79 - 40.38) | NA | NA | 85.89 |
|  | Consensus standard | Pilotte qPCR | 6 | 32 | 10 | 406 | 15.79 (4.20 - 27.38) | 97.60 (96.12 - 99.07) | 37.50 | 92.69 |
|  |  | Llewellyn qPCR | 23 | 15 | 12 | 394 | 60.53 (44.99 - 76.07) | 97.04 (95.40 - 98.69) | 65.71 | 96.33 |
|  |  | Verweij qPCR | 34 | 4 | 18 | 388 | 89.47 (79.72 - 99.23) | 95.57 (93.56 - 97.57) | 65.38 | 98.98 |
|  |  | Chankongsin qPCR | 33 | 5 | 5 | 401 | 86.84 (76.09 - 97.59) | 98.77 (97.70 - 99.84) | 86.84 | 98.77 |
|  |  | Watts LAMP | 24 | 14 | 2 | 404 | 63.16 (47.82 - 78.49) | 99.51 (98.83 - 100) | 92.31 | 96.65 |
| **Gabon** | Composite standard | Pilotte qPCR | 10 | 45 | 0 | 186 | 18.18 (7.99- 28.38) | NA | NA | 80.52 |
|  |  | Llewellyn qPCR | 18 | 37 | 0 | 186 | 32.73 (20.33 - 45.13) | NA | NA | 83.41 |
|  |  | Verweij qPCR | 38 | 17 | 0 | 186 | 69.09 (56.88 - 81.30) | NA | NA | 91.63 |
|  |  | Chankongsin qPCR | 18 | 37 | 0 | 186 | 32.73 (20.33 - 45.13) | NA | NA | 83.41 |
|  |  | Watts LAMP | 13 | 42 | 0 | 186 | 23.64 (12.41 - 34.86) | NA | NA | 81.58 |
|  | Consensus standard | Pilotte qPCR | 3 | 19 | 7 | 212 | 13.64 (0.00 - 27.98) | 96.80 (94.47 - 99.13) | 30.00 | 91.77 |
|  |  | Llewellyn qPCR | 10 | 12 | 8 | 211 | 45.45 (24.65 - 66.26) | 96.35 (93.86 - 98.83) | 55.56 | 94.62 |
|  |  | Verweij qPCR | 22 | 0 | 16 | 203 | 100 (NA) | 92.69 (89.25 - 96.14) | 57.89 | 100.00 |
|  |  | Chankongsin qPCR | 17 | 5 | 1 | 218 | 77.27 (59.76 - 94.78) | 99.54 (98.65 - 100) | 94.44 | 97.76 |
|  |  | Watts LAMP | 12 | 10 | 1 | 218 | 54.55 (33.74 - 73.35) | 99.54 (98.65 - 100) | 92.31 | 95.61 |
| **Papua New Guinea** | Composite standard | Pilotte qPCR | 6 | 24 | 0 | 186 | 20.00 (5.69 - 34.31) | NA | NA | 88.57 |
|  |  | Llewellyn qPCR | 17 | 13 | 0 | 186 | 56.67 (38.93 - 74.40) | NA | NA | 93.47 |
|  |  | Verweij qPCR | 14 | 16 | 0 | 186 | 46.67 (28.81 - 64.52) | NA | NA | 92.08 |
|  |  | Chankongsin qPCR | 20 | 10 | 0 | 186 | 66.67 (49.80 - 83.54) | NA | NA | 94.90 |
|  |  | Watts LAMP | 13 | 17 | 0 | 186 | 43.33 (25.60 - 61.07) | NA | NA | 91.63 |
|  | Consensus reference standard | Pilotte qPCR | 3 | 13 | 3 | 184 | 18.75 (0.00 - 37.87) | 98.40 (96.59 - 100) | 50.00 | 93.40 |
|  |  | Llewellyn qPCR | 13 | 3 | 4 | 183 | 81.25 (62.13 - 100) | 97.86 (95.79 - 99.93) | 76.47 | 98.39 |
|  |  | Verweij qPCR | 12 | 4 | 2 | 185 | 75.00 (53.78 - 96.22) | 98.93 (97.46 - 100) | 85.71 | 97.88 |
|  |  | Chankongsin qPCR | 16 | 0 | 4 | 183 | 100 (NA) | 97.86 (95.79 - 99.93) | 80.00 | 100.00 |
|  |  | Watts LAMP | 12 | 4 | 1 | 186 | 75.00 (53.78 - 96.22) | 99.47 (98.42 - 100) | 92.31 | 97.89 |

TP – true positive; FN – false negative; FP – false positive; TN – true negative; 95% CI – 95% confidence interval; PPV – positive predictive value, NPV – negative predictive value
