# Supplementary material for: Five ways to find a parasite: comparison of five molecular assays for the detection of Strongyloides spp
Source: J Clin Microbiol. 2026 Oct 12;64(11):e00137-26. doi: 10.1128/jcm.00137-26 (PMC13753838; doi:10.1128/jcm.00137-26)
Supplement: Supplementary file 5 [file jcm.00137-26-s0005.docx]

**Supplementary table 2:** Results of sequencing assay amplicons for stool samples from Papua New Guinea showing average Ct scrore, assay results (+/-), and result of sequencing analysis with the percentage identity and coverage (%/%).

|  | **Pilotte** | | | **Llewellyn** | | | **Chankongsin** | | | **Verweij** | | | **LAMP** | |
| --- | --- | --- | --- | --- | --- | --- | --- | --- | --- | --- | --- | --- | --- | --- |
| **Sample** | **Ct average** | **P/N** | **Sequencing** | **Ct average** | **P/N** | **Sequencing** | **Ct average** | **P/N** | **Sequencing** | **average** | **P/N** | **Sequencing** | **P/N** | **Sequencing** |
| 1 | NA | - |  | 35.53 | + | **did not seq** | NA | - |  | NA | - |  | - |  |
| 17 | NA | - |  | NA | - |  | NA | - |  | NA | - |  | - |  |
| 21 | NA | - |  | NA | - |  | NA | - |  | NA | - |  | - |  |
| 32 | NA | - |  | 27.40 | + | S. fuelleborni (100% / 99.6%) | 32.10 | + | Mediterraneibacter gnavus (98.4% / 98.4%)) | 33.02 | + | **did not seq** | + | S. procyonis, S. robustus (100%), S. stercoralis (99.4%) |
| 41 | NA | - |  | NA | - |  | 37.63 | + | Mediterraneibacter gnavus (98.73 / 98.73%) | NA | - |  | - |  |
| 57 | NA | - |  | NA | - |  | 36.11 | + | **did not seq** | NA | - |  | + | **did not seq** |
| 60 | NA | - |  | 29.23 | + | S. fuelleborni (100% / 99.6%) | 32.48 | + | **did not seq** | 32.65 | + | **did not seq** | + | **did not seq** |
| 82 | NA | - |  | NA | - |  | NA | - |  | 36.40 | + | S. stercoralis (100% / 100%) |  |  |
| 84 | NA | - |  | NA | - |  | 38.46 | + | Bacteriophage (99.8% / 99.8%) | NA | - |  | - |  |
| 95 | NA | - |  | 33.73 | + | S. fuelleborni (99.75% / 99.5%) | 38.96 | + | **did not seq** | NA | - |  | - |  |
| 107 | NA | - |  | 28.96 | + | S. fuelleborni (99.5% / 99.5%) | 31.75 | + | Mediterraneibacter gnavus (98.9% / 98.9%) | NA | - |  | + | S. procyonis , S. robustus (100% / 100%), S. stercoralis (99.4% / 99.4%) |
| 124 | 28.23 | + | S. stercoralis (100% / 100%) | NA | - |  | NA | - |  | NA | - |  | - |  |
| 130 | 34.60 | + | S. stercoralis (98.56% / 100%) | 31.26 | + | S. ransomi (100% / 100%) | 34.26 | + | **did not seq** | 33.63 | + | S. stercoralis (98% / 100%) | + | S. procyonis , S. robustus (100% / 100%), S. stercoralis (99.4% / 99.4%) |
| 133 | NA | - |  | NA | - |  | NA | - |  | NA | - |  | + | **did not seq** |
| 137 | NA | - |  | 28.68 | + | S. fuelleborni (100% / 99.4%) | 30.78 | + | **did not seq** | 29.59 | + | **did not seq** | + | S. procyonis, S. robustus (100%), S. stercoralis (99.4%) |
| 150 | NA | - |  | 35.31 | + | S. fuelleborni (99.5% / 99.5%) | 36.63 | + | Mediterraneibacter gnavus (99.7% / 99.7%) | NA | - |  | - |  |
| 11M | NA | - |  | 39.17 | - |  | 38.57 | + | **did not seq** | NA | - |  | - |  |
| 16M | 36.66 | - |  | NA | - |  | NA | - |  | NA | - |  | - |  |
| 17M | NA | - |  | NA | - |  | 37.25 | + | **did not seq** | 27.03 | + | **did not seq** | - |  |
| 18C | NA | - |  | NA | - |  | 31.33 | + | **did not seq** | 30.88 | + | **did not seq** | + | S. procyonis , S. robustus (100% / 100%), S. stercoralis (99.3% / 99.3%) |
| 18M | 33.62 | + | S. stercoralis (87% / 96.34%) | 36.47 | + | S. stercoralis (100% / 99.9%) | 32.80 | + | **did not seq** | 36.50 | + | **did not seq** | + | S. stercoralis (100% / 100%) |
| 19M | 36.53 | - |  | NA | - |  | NA | - |  | NA | - |  | - |  |
| 1M | 35.87 | + | S. stercoralis (99.3% / 99.3%) | NA | - |  | NA | - |  | NA | - |  | - |  |
| 23M | NA | - |  | 36.49 | + | S. stercoralis (100% / 100%) | 38.19 | + | **did not seq** | 35.57 | + | **did not seq** | - |  |
| 24M | NA | - |  | NA | - |  | 32.94 | + | **did not seq** | NA | - |  | - |  |
| 29M | NA | - |  | 38.80 | + | S. stercoralis (100% / 100%) | 32.67 | + | **did not seq** | 36.26 | + | **did not seq** | + | S. stercoralis (99.4% / 99.4%) |
| 2M | 32.63 | + | S. stercoralis (100% / 100%) | 33.51 | + | S. stercoralis (100% / 100%) | 31.64 | + | **did not seq** | 33.95 | + | **did not seq** | + | S. stercoralis (100% / 100%) |
| 31M | NA | - |  | NA | - |  | NA | - |  | 31.54 | + | **did not seq** | - |  |
| 4C | NA | - |  | 31.93 | + | S. fuelleborni (100% / 99.6%) | 32.37 | + | **did not seq** | 32.98 | + | **did not seq** | + | S. procyonis, S. robustus (99.4%), S. stercoralis (98.9%) |
| 4M | 37.55 | + | S. stercoralis (100% / 100%) | NA | - |  | NA | - |  | NA | - |  | - |  |
| 5M | NA | - |  | 37.09 | + | S. stercoralis (68.63% / 100%) | NA | - |  | NA | - |  | - |  |
| 7M | NA | - |  | 37.20 | + | S. stercoralis (69.80% / 100%) | NA | - |  | NA | - |  | - |  |
| 8C | NA | - |  | 26.62 | + | S. fuelleborni (100% / 00/6%) | 29.49 | + | **did not seq** | 29.70 | + | **did not seq** | + | S. procyonis (100%), S. stercoralis (99.4%) |
| 8M | NA | - |  | 36.13 | + | S. stercoralis (99.5% / 99.3%) | NA | - |  | NA | - |  | - |  |
