# Supplementary material for: Five ways to find a parasite: comparison of five molecular assays for the detection of Strongyloides spp
Source: J Clin Microbiol. 2026 Oct 12;64(11):e00137-26. doi: 10.1128/jcm.00137-26 (PMC13753838; doi:10.1128/jcm.00137-26)
Supplement: Supplementary file 6 [file jcm.00137-26-s0006.docx]

**Supplementary table 3:** Results of sequencing assay amplicons for stool samples from Gabon showing average Ct scrore, assay results (+/-), and result of sequencing analysis with the percentage identity and coverage (%/%).

|  | **Pilotte** | | | | **Llewellyn** | | | **Chankongsin** | | | **Verweij 18S (<35)** | | | **LAMP** | | |
| --- | --- | --- | --- | --- | --- | --- | --- | --- | --- | --- | --- | --- | --- | --- | --- | --- |
| **Sample** | **Ct average** | **P/N** | **Sequencing** | **Ct average** | | **P/N** | **Sequencing** | **Ct average** | **P/N** | **Sequencing** | **Ct average** | **P/N** | **Sequencing** | **P/N** | **Sequencing** |  |
| 13 | 35.96 | + | S. stercoralis (100% / 100%) | 38.34 | | - |  | NA | - |  | NA | - |  | - |  |  |
| 15 | 36.93 | - |  | 35.615 | | + | S. fuelleborni (99.6% / 99.4%) | 36.85 | + | S. fuelleborni, S. planiceps, S. cebus, S. robustus, S. callosciureus (100% / 100%) | 30.72 | + | **Did not seq** | **+** | S. procyonis/S. stercoralis/S. ratti (100% / 100%) |  |
| 18 | 37.74 | - |  | 36.48 | | + | S. fuelleborni (100% / 100%) | 36.42 | + | Anaerostipes hadrus (98.3% / 98.3%) | 32.73 | + | **Did not seq** | + | S. fuelleborni (100% / 100%) |  |
| 21 | 38.05 | - |  | NA | | - |  | 38.12 | - |  | NA | - |  |  |  |  |
| 23 | 35.70 | + | S. stercoralis (100% / 100%) | NA | | **-** |  | **39.1** | **+** | **Did not seq** | 33.31 | + | **Did not seq** | **-** |  |  |
| 25 | NA | - |  | 37.89 | | + | S. stercoralis (100% / 100%) | NA | - |  | NA | - |  |  |  |  |
| 26 | 37.20 | - |  | 36.93 | | - |  | NA | - |  | 34.20 | + | Segatella copri (96.2% / 96.2%) |  |  |  |
| 27 | 38.70 | - |  | 37.355 | | + | S. papillosus / S.fuelleborni (98.4% / 98.4%) | NA | - |  | 34.08 | + | **Did not seq** |  |  |  |
| 28 | 38.40 | - |  | NA | | - |  | NA | - |  | 33.70 | + | **Did not seq** |  |  |  |
| 35 | 31.93 | + | S. stercoralis (100% / 100%) | 35.35 | | + | S. stercoralis (100% / 100%) | NA | - |  | 32.97 | + | S. stercoralis (44.1% / 92.9%) | - |  |  |
| 38 | 39.59 | - |  | NA | | - |  | NA | - |  | NA | - |  |  |  |  |
| 47 | NA | - |  | 37.25 | | + | S. stercoralis (99,6% / 99.6%) | NA | - |  | NA | - |  |  |  |  |
| 50 | 37.42 | - |  | 39.84 | | - |  | NA | - |  | NA | - |  | + | **Did not seq** |  |
| 55 | 38.04 | - |  | NA | | - |  | NA | - |  | NA | - |  |  |  |  |
| 60 | 36.25 | - |  | NA | | - |  | NA | - |  | NA | - |  |  |  |  |
| 63 | 36.22 | - |  | NA | | - |  | NA | - |  | 38.01 | - |  | - |  |  |
| 67 | 35.54 | + | S. stercoralis (100% / 100%) | 36.95 | | - |  | NA | - |  | NA | - |  | - |  |  |
| 71 | 36.91 | - |  | NA | | - |  | NA | - |  | 34.28 | + | S. stercoralis (95.9% / 95/9%) | - |  |  |
| 75 | NA | - |  | 37.96 | | + | S. stercoralis (100% / 100%) | NA | - |  | NA | - |  | - |  |  |
| 78 | NA | - |  | 37.97 | | + | S. ransomi /S. stercoralis 99.2% | NA | - |  | NA | - |  | - |  |  |
| 79 | NA | - |  | 37.76 | | + | S. stercoralis (100% / 100%) | NA | - |  | NA | - |  | - |  |  |
| 80 | NA | - |  | 37.96 | | + | S. stercoralis (100% / 100%) | NA | - |  | NA | - |  | - |  |  |
| 88 | 35.88 | + | S. stercoralis (100% / 100%) | 38.61 | | - |  | NA | - |  | NA | - |  | - |  |  |
| 89 | 36.90 | - |  | 31.79 | | + | S. ransomi/S. stercoralis (99.3% / 99.3%) | NA | - |  | NA | - |  | - |  |  |
| 91 | 37.76 | - |  | NA | | - |  | NA | - |  | 34.82 | + | Segatella copri (97.6% / 97.4%) | - |  |  |
| 94 | 38.72 | - |  | NA | | - |  | NA | - |  | NA | - |  | - |  |  |
| 95 | 38.79 | - |  | 36.53 | | + | S. stercoralis (100% / 100%) | 31.73 | + | Segatella copri (97.9% / 97.9%) | 30.56 | + | Segatella copri (98.8% / 98.8%) | + | S. stercoralis (99.7% / 97.7%) |  |
| 96 | 38.92 | - |  | NA | | - |  | NA | - |  | 33.59 | + | **Did not seq** | - |  |  |
| 97 | 38.68 | - |  | NA | | - |  | NA | - |  | NA | - |  | - |  |  |
| 102 | 37.99 | - |  | NA | | - |  | NA | - |  | 35.64 | - |  | - |  |  |
| 106 | 36.54 | + | **Did not seq** | 38.23 | | - |  | NA | - |  | NA | - |  | - |  |  |
| 108 | 36.45 | - |  | 37.53 | | - |  | 38.1 | - | Segatella copri 97.1% identity | 31.59 | + | **Did not seq** | + | **Did not seq** |  |
| 115 | 37.10 | - |  | 34.63 | | + | S. fuelleborni (100% / 100%) | 32.58 | + | **Did not seq** | 26.16 | + | **Did not seq** | + | S. stercoralis (97.8% / 97.8%) |  |
| 116 | 37.60 | - |  | 37.42 | | - |  | 33.96 | + | E. coli (97.7% / 97.7%) | 28.16 | + | **Did not seq** | + | **Did not seq** |  |
| 118 | 37.40 | - |  | 36.46 | | + | S. fuelleborni (100% / 100%) | 33.94 | + | Segatella copri (97.1% / 97.1%) | 32.72 | + | **Did not seq** | + | S. procyonis/S. stercoralis/S. ratti (100% / 100%) |  |
| 119 | 37.41 | - |  | 38.24 | | - |  | 35.76 | + | segatella copri (98.5% / 98.5%) | 30.10 | + | **Did not seq** | - |  |  |
| 120 | 37.10 | - |  | 37.98 | | - |  | 38.7 | + | segatella copri 98.5% | 31.64 | + | **Did not seq** | - |  |  |
| 121 | 37.94 | - |  | 37.19 | | - |  | 35.77 | + | **Did not seq** | 27.52 | + | **Did not seq** | + | S. stercoralis/S. ratti (100% / 100%) |  |
| 122 | 37.55 | - |  | NA | | - |  | NA | - |  | 32.60 | + | **Did not seq** | **-** |  |  |
| 124 | 37.41 | **-** |  | **32.23** | | **+** | **S. fuelleborni (99.3% / 99.1%)** | **30.54** | **+** | **Did not seq** | 23.16 | + | **Did not seq** | + | **Did not seq** |  |
| 130 | 35.71 | + | S. stercoralis (99.0% / 99.0%) | 38.39 | | - |  | NA | - |  | NA | - |  | - |  |  |
| 152 | 38.05 | - |  | NA | | - |  | NA | - |  | 34.77 | + | **Did not seq** | - |  |  |
| 162 | 38.76 | - |  | NA | | - |  | NA | - |  | 34.81 | + | **Did not seq** | - |  |  |
| 170 | 36.83 | - |  | NA | | - |  | NA | - |  | 34.55 | + | **Did not seq** | - |  |  |
| 174 | 32.53 | + | **S. stercoralis (100% / 100%)** | 38.93 | | - |  | NA | - |  | NA | - |  | - |  |  |
| 181 | 36.71 | - |  | NA | | - |  | NA | - |  | NA | - |  | - |  |  |
| 183 | 38.34 | - |  | 38.11 | | - |  | 38.06 | + | segatella copri (98.9% / 98.9%) | 32.59 | + | **Did not seq** | - |  |  |
| 184 | 37.86 | - |  | NA | | - |  | NA | - |  | NA | - |  | - |  |  |
| 186 | 37.61 | - |  | NA | | - |  | NA | - |  | 34.58 | + | **Did not seq** | - |  |  |
| 192 | 36.28 | - |  | 37.61 | | - |  | 39.1 | + | **Did not seq** | NA | - |  | - |  |  |
| 193 | 36.97 | - |  | 38.1 | | - |  | 35.21 | + | **Did not seq** | 33.05 | + | **Did not seq** | + | **Did not seq** |  |
| 195 | 36.03 | - |  | NA | | - |  | NA | - |  | 33.49 | + | S. stercoralis (100% / 100%) | - |  |  |
| 208 | 38.16 | - |  | NA | | - |  | NA | - |  | 34.32 | + | Segatella copri (97.2 / 97.2%) | - |  |  |
| 212 | 37.43 | - |  | NA | | - |  | NA | - |  | 34.96 | + | **Did not seq** | - |  |  |
| 213 | 37.98 | - |  | NA | | - |  | 38.95 | + | Segatella copri (94.9% / 94.9%) | 34.09 | + | **Did not seq** | - |  |  |
| 215 | 37.12 | - |  | NA | | - |  | NA | - |  | NA | - |  | - |  |  |
| 219 | 37.27 | - |  | 37.41 | | - |  | 38.5 | + | Mediterraneibacter gnavus (98.9% / 98.9%) | 30.14 | + | S. stercoralis, S. ratti, S. procyonis etc (100% / 100%) | - |  |  |
| 220 | 37.15 | - |  | NA | | - |  | NA | - |  | 34.17 | + | **Did not seq** | - |  |  |
| 222 | NA | - |  | 37.98 | | + | S. stercoralis (100% / 100%) | NA | - |  | NA | - |  | - |  |  |
| 225 | 35.91 | + | S. stercoralis (100% / 100%) | NA | | - |  | 39.05 | - |  | NA | - |  | - |  |  |
| 226 | 37.28 | - |  | 37.97 | | + | S. stercoralis (100% / 100%) | NA | - |  | 33.78 | + | S. stercoralis (100% / 100%) | - |  |  |
| 229 | 36.23 | - |  | 34.71 | | + | S. fuelleborni (100% / 100%) | 34.51 | + | Segatell copri (100% / 100%) | 30.92 | + | S. stercoralis (100% / 100%) | + | **Did not seq** |  |
| 233 | 36.55 | - |  | NA | | - |  | NA | - |  | 33.53 | + | **Did not seq** | - |  |  |
| 237 | 36.34 | - |  | NA | | - | ` | NA | - |  | 36.71 | - |  | - |  |  |
| 238 | 33.57 | + | S. stercoralis (100% / 100%) | 36.05 | | - |  | 39.3 | - |  | 33.90 | + | **Did not seq** | - |  |  |
| 241 | 36.82 | - |  | NA | | - |  | NA | - |  | 34.12 | + | **Did not seq** | - |  |  |
| 4B | 36.34 | - |  | NA | | - |  | 24.87 | + | **Did not seq** | **33.30** | + | **Did not seq** | + | S. stercoralis (100% / 100%) |  |
