# Supplementary material for: Five ways to find a parasite: comparison of five molecular assays for the detection of Strongyloides spp
Source: J Clin Microbiol. 2026 Oct 12;64(11):e00137-26. doi: 10.1128/jcm.00137-26 (PMC13753838; doi:10.1128/jcm.00137-26)
Supplement: Supplementary file 7 [file jcm.00137-26-s0007.docx]

**Supplementary table 4:** e-values of primers and proves from each assay when restricted to 1) *Segatella copri*, 2) *Anaerostipes hadrus*, 3) *Mediterraneibacter gnavus*, 4) no restrictions, and 5) with *Strongyloides* excluded.

| **Assay** | **F/R/P** | **e-value** | **Restricted to species** | **e-value** | **Restricted to species** | **e-value** | **Restricted to species** | **No restriction (query coverage / identity)** | ***Strongyloides e*xcluded (query coverage / identity)** |
| --- | --- | --- | --- | --- | --- | --- | --- | --- | --- |
| Chankongsin qPCR | F | 0.002 | *Segatella copri* | 0.055 | *Anaerostipes hadrus* | *0.43* | *Mediterraneibacter gnavus* | Various *Strongyloides* species (100% / 100%) | *Halechiniscus churakaagii* (100% / 100%) |
|  | R | 0.39 |  | 2.1 |  | 4.3 |  | Various *Strongyloides* species (100% / 100%) | *Vulpes vulpes* (91% / 100% ) |
|  | P | 1.5 |  | 0.034 |  | 5.6 |  | Various *Strongyloides* species (100% / 100%) | *Helicotylenchus multicinctus* (92% / 100%) |
| Pilotte qPCR | F | 6.1 |  | 2.1 |  | 17 |  | Only repeat for *S. stercoralis* (100% / 100%) | *Geotrypetes seraphini* (91% / 100%) |
|  | R | 0.098 |  | 8.2 |  | 67 |  | Only repeat for *S. stercoralis* (100% / 100%) | Mouse (82% / 100%) |
|  | P | 2.3 |  | 0.86 |  | 6.8 |  | Only repeat for *S. stercoralis* (100% / 100%) | *Pteropus vampyrus* (78% / 100%) |
| Llewellyn qPCR | F | 1.2 |  | 6.2 |  | 1.2 |  | Various strongy species (100% / 100%) | *Rhabditida* sp. (100% / 100%) |
|  | R | 0.39 |  | 0.59 |  | 1.2 |  | Various *Strongyloides* species (100% / 100%), note, not 100% for *S. fuelleborni* | *Corybacterium kutscheri* (83% / 100%) |
|  | P | 15 |  | 24 |  | 152 |  | Various *Strongyloides* species, as well as *Meloidogune paranaensis, Acrobeloides, Streptomyces aurantiacus*, predicted *Bacillus rossius redtenbacheri* (100% / 100%) | *Meloidogune paranaensis, Acrobeloides, Streptomyces aurantiacus, uncultured eukaryoite clone,* predicted *Bacillus rossius redtenbacheri* (100% / 100%) |
| Verweij qPCR | F | 2.5 |  | 3.6 |  | 29 |  | Various strongy species (100% / 100%) | *Parastrongyloides trichosuri* (100% / 96.43%) |
|  | R | 0.39 |  | 0.59 |  | 1.2 |  | Various *Strongyloides* species (100% / 100%), note, not 100% for *S. fuelleborni* | *Corybacterium kutscheri* (83% / 100%) |
|  | P | 15 |  | 24 |  | 152 |  | Various *Strongyloides* species, as well as *Meloidogune paranaensis, Acrobeloides, Streptomyces aurantiacus*, predicted *Bacillus rossius redtenbacheri* (100% / 100%) | *Meloidogune paranaensis, Acrobeloides, Streptomyces aurantiacus,* predicted *Bacillus rossius redtenbacheri* (100% / 100%) |
| Watts LAMP | F3 | 2.3 |  | 16 |  | 114 |  | Various *Strongyloides* species and, *Macaca nemestrina, Meanogrammus aeflefinus, Leishmania mexicana, Chlamydomonas reinhardtii, Exophiala mesophila, Actinidia erithantha, Microbulbifer* (100% / 100%) | *Macaca nemestrina, Meanogrammus aeflefinus, Leishmania mexicana, Chlamydomonas reinhardtii, Exophiala mesophila, Actinidia erithantha, Microbulbifer* |
|  | B3 | 5.3 |  | 7.2 |  | 3.7 |  | Various *Strongyloides* species (100% / 100%) | *Medicago truncatula* (90% / 100%) |
